# Feasibility and Acceptability of a Digital Indoor Air Quality Monitor Among Children with Asthma

**DOI:** 10.2196/92070

**Published:** 2026-08-07

**Authors:** Olivia Orr, Kyle Honegger, Lizbeth Sanchez, Kiran Bhat, Sai Nimmagadda, Kristin Kan

**Affiliations:** 1 Mary Ann & J. Milburn Smith Child Health Outcomes, Research, and Evaluation Center, Ann and Robert H. Lurie Children's Hospital of Chicago, 225 E Chicago Ave, Chicago, IL, 60611, United States, 1 3122276110; 2 Stanley Manne Children's Research Institute, Ann and Robert H. Lurie Children's Hospital of Chicago, Chicago, IL, United States; 3 DePaul University, Chicago, IL, United States; 4 Northwestern University, Evanston, IL, United States; 5 Center for Food Allergy and Asthma Research, Northwestern University Feinberg School of Medicine, Chicago, IL, United States; 6 Division of Pediatric Allergy and Immunology, Department of Pediatrics, Northwestern University Feinberg School of Medicine, Chicago, IL, United States; 7 Division of Advanced General Pediatrics & Primary Care, Department of Pediatrics, Northwestern University Feinberg School of Medicine, Chicago, IL, United States

**Keywords:** air quality monitor, pediatric asthma, feasibility, acceptability, implementation, digital monitoring

## Abstract

This pilot study examined the feasibility and acceptability of implementing a digital in-home air quality monitor among families with children with asthma (N=20). Interview and survey data indicated that the use of this tool was both feasible and acceptable, while highlighting affordability as an important concern for use in future clinical practice for asthma management.

## Introduction

Approximately 6 million US children have asthma, and between school and home, they spend 80%-90% of the day indoors [1]. There is evidence that indoor air exposure significantly affects asthma severity. Studies emphasize that the indoor environment and housing quality are crucial determinants of asthma outcomes, yet self-reported assessments are subject to recall bias and misclassification of exposures [2]. The emergence of digital indoor air quality monitors offers a possible scalable and sustainable approach to indoor air data collection that could aid in tailored recommendations for the home environment [3,4]. However, studies utilizing these monitors are still limited in clinical care, and concerns remain about equitable deployment of these tools [3]. The aims of this study are (1) to assess the feasibility and acceptability of using digital in-home air quality monitors in the homes of children with asthma and (2) evaluate the feasibility of providing a WiFi hotspot to overcome internet connectivity barriers.

## Methods

### Study Design

This was a pilot feasibility study with 20 participants recruited from a pediatric pulmonary clinic from December 2023 to March 2024. Eligibility criteria included (1) children aged 4-17 years old, (2) the *ICD-10* (*International Classification of Diseases, Tenth Revision*) code for persistent asthma in the past 12 months, (3) sleeping in the same room of the same house for 4 or more nights a week (ie, the child’s bedroom), and (4) English-speaking caregivers.

The study intervention included an air quality monitor (uHoo Smart Air Monitor) that was placed in the child’s bedroom for 3 months, a pre-paid WiFi hotspot (Simple Mobile Moxee Wi-Fi Hotspot), and a summary review with a pediatric allergist at the end of the study. Relative to commercial grade monitors, the uHoo air quality monitor is marketed directly to consumers and may be a more affordable option for some households (US $300). It uses sensors with auto-calibration algorithms to obtain measurements of 8 air quality parameters: carbon monoxide, carbon dioxide, nitrogen dioxide, ozone, particulate matter 2.5, relative humidity, total volatile organic compounds, and temperature [5]. This device was chosen because of its cost and its acceptable stability and calibration, measured in a laboratory [5]. The WiFi hotspot was paired with the air quality monitor for internet connectivity to upload the data to the uHoo web portal. Participants did not have access to the uHoo app or portal that displayed real-time data and were informed at enrollment that aggregated data would only be reviewed at the completion of the study.

Baseline surveys at enrollment inquired about demographics, home environment, asthma control, digital literacy (ranging from 0-12, with higher scores indicating higher literacy) [6], and asthma self-management (ranging from 0-5, with higher scores indicating higher self-efficacy) [7]. Exit surveys, completed 3 months from enrollment, assessed asthma control, feasibility, acceptability, and usability with the System Usability Scale (SUS) [8]. Feasibility was defined a priori as 1 week of continuous data collection by monitors. Acceptability was assessed through the brief exit semistructured interview and survey. SUS scores range from 1-100, with 70 indicating an “acceptable” level of usability. After the exit survey, a semistructured interview was completed by televideo, and before the interview, the pediatric allergist reviewed the summary air quality findings from the monitor, the child’s asthma control scores, and home practices to improve air quality.

Descriptive statistics included frequencies, proportions, and median and IQRs for sample characteristics and survey responses. Interviews were audio-recorded and transcribed, and a deductive approach was used for qualitative analysis (coders: LS, KB, OO, KK) regarding feasibility and acceptability. A convergent mixed methods approach was used to connect interview responses with the survey data.

### Ethical Considerations

The Ann & Robert H. Lurie Children’s Hospital of Chicago Institutional Review Board (IRB 2023‐6409) approved this study. The institutional review board determined that this study involves no greater than minimal risk.

## Results

The study sample was mostly composed of mothers (n=17, 85%), those living in an apartment/duplex (n=13, 65%), and those with a high school diploma or less (n=12, 60%) (Table 1). Baseline digital health literacy and asthma management self-efficacy were high.

**Table 1.** Sample characteristics (N=20).

	Value
Caregiver age, median (IQR)	39.5 (34.75-43.25)
Caregiver sex, n (%)	
Female	18 (90)
Male	2 (10)
Other	0 (0)
Caregiver race/ethnicity, n (%)	
Hispanic/Latino	7 (35)
Black/African American	7 (35)
White	5 (25)
Asian	1 (5)
Caregiver relationship to child, n (%)	
Parent (mother or father)	19 (95)
Grandparent	1 (5)
Caregiver education, n (%)	
High school diploma or less	12 (60)
More than a high school diploma	8 (40)
Household income (US $), n (%)	
Less than 30,000	9 (45)
30,000-74,999	6 (30)
75,000 and above	5 (25)
Home type, n (%)	
Apartment/duplex	13 (65)
Single family	5 (25)
Condominium/townhouse	1 (5)
Other	1 (5)
Child age, median (IQR)	7 (5.5-11)
Child sex, n (%)	
Female	7 (35)
Male	13 (65)
Other	0 (0)
Child race/ethnicity, n (%)	
Hispanic/Latino	8 (40)
Black/African American	7 (35)
White	4 (20)
Asian	1 (5)
Baseline DHLQ^a^, median (IQR)	12 (10.75-12)
Baseline PAMSES^b^, median (IQR)	
Attack prevention self-efficacy	4.8 (4.3-5)
Exacerbation management self-efficacy	4.6 (4.25-5)
ACT/c-ACT^c^, median (IQR**)**	
Baseline	19 (18-21)
Final (3-month survey)	20 (17.5-21.5)

a DHLQ: Digital Health Literacy Questionnaire.

b PAMSES: Parental Asthma Management and Self-Efficacy Scale.

c Asthma Control Test (ACT) and Child Asthma Control Test (c-ACT) were combined due to small sample size.

### Feasibility

Nineteen of 20 enrolled participants successfully collected continuous indoor air quality data for at least 1 week and averaged 81 (SD 19) out of 90 days. One participant never completed set-up of the device at home. Indoor air data collected an average of 72% (SD 16%) of the available hours for each participant, ranging from 34% to 97%, and 74% of available days had at least 12 hours of data collection. Feasibility measures were rated highly (Table 2 and Multimedia Appendix 1).

**Table 2.** Feasibility and acceptability of using a digital indoor air quality monitor.^a^

	Value, n (%)
Feasibility survey questions (n=15)	
How often did the child sleep in the same room as the air quality monitor?	
Everyday	15 (100)
>50% of days in a week (4 or more)	0 (0)
<50% of days in a week (3 or less)	0 (0)
How would you rate the set-up of the air quality monitor (uHoo- the white device) at home?^b^	
Easy/very easy	15 (100)
How would you rate the set-up of the Wi-Fi hotspot at home (the black device?)^b^	
Easy/very easy	15 (100)
Acceptability survey questions (n=15)	
How likely are you to use the air quality monitor in the future if this device was provided to you in clinic?^c^	
Neutral	2 (13)
Very likely/likely	13 (87)
How likely are you to recommend this technology to a family caring for a child with asthma?^c^	
Neutral	2 (13)
Very likely/likely	13 (87)
Would you have joined the study if the WiFi hotspot was not provided?	
Yes	11 (73)
No	1 (7)
Not sure	3 (20)

a All participants who completed the exit interview (n=13) had corresponding exit survey data.

b Other item responses included very difficult and difficult or neutral.

c Other item responses included very unlikely and unlikely.

### Acceptability

Fifteen of 20 enrolled participants completed exit surveys and 13 completed exit interviews. Most participants rated acceptability questions highly (Table 2). While most participants reported they would have joined the study if the WiFi hotspot was not provided, only half interviewed (n=7) stated that they would use the monitors if they had to pay for it. Participants described that the “price may be prohibitive” and “insurance probably should cover” it. On the SUS, participants rated the intervention with a median of 67.5 (IQR 56.3‐81.3).

## Discussion

This study demonstrated that data collection with digital indoor air quality was feasible and acceptable to a socioeconomically varied sample of families with children with asthma.

The high feasibility may be due to the pre-pairing of monitors with WiFi hotspots so that participants only had to plug in each device at home with no additional set-up. The air monitor intervention’s usability score, despite scoring similarly to other air pollution monitors (SUS: 50-66)*,* fell slightly below the threshold for acceptable usability, suggesting further inquiry is needed to understand how usability can be improved. Nevertheless, this rating was similar to how the public rated using cellphones in past evaluations (SUS: 66.5) [8]. Nevertheless, over a quarter of participants were unsure if they would have joined without the WiFi hotspot. Proponents of health equity have highlighted that neighborhood broadband access and perceived digital self-efficacy can limit access and use of digital health tools [9].

For acceptability, most participants reported a future interest in using a digital air monitor if provided by the clinic, and the data reviewed with the pediatric allergist were well-received. This finding fits with those from other digital health studies, that is, that closed-loop communication by the health care team about patient-generated health data is crucial to enhance chronic disease self-management [10]. However, this study’s aggregated data report, short enrollment period, and device limitations prohibited more tailored recommendations for improving asthma control.

Despite interest in the monitors, half of the participants also identified that they could not afford the cost of the monitor themselves. A recent Centers for Medicare & Medicaid Services (CMS) policy will address this cost barrier to digital health adoption by reimbursing technology-supported health care, including wearable devices and health coaching apps, for Medicare-insured patients with certain chronic conditions [11]. While this CMS model may alleviate costs for some, this will not address affordability for pediatric populations, and expanded access models for technology-supported pediatric health care should be considered.

Study limitations included (1) participants’ lack of real-time air quality data during the study, which affected their ability to make at-home modifications, (2) the inability of this type of air monitor to detect indoor environmental allergens (eg, mold, dander), and (3) the short enrollment period and small sample size, which limited inferences about sustained engagement with the air monitors and impact on asthma control, respectively.

Though the feasibility and acceptability of the digital air quality monitoring intervention were promising in this study, affordability was highlighted by participants as a key barrier to the use of these tools in clinical care. Future studies should also incorporate real-time feedback and associated clinical management with air quality monitoring year-round.

## Supplementary material

10.2196/92070 Multimedia Appendix 1 Survey questions and interview findings of the feasibility and acceptability of using a digital indoor air quality monitor.

## Abbreviations

CMS: Centers for Medicare & Medicaid Services
*ICD-10*: *International Classification of Diseases, Tenth Revision*
SUS: System Usability Scale
