# Supplementary material for: Feasibility and Acceptability of a Digital Indoor Air Quality Monitor Among Children with Asthma
Source: JMIR Pediatr Parent. 2026 Aug 7;9:e92070. doi: 10.2196/92070 (PMC13450806; doi:10.2196/92070)
Supplement: Supplementary file 1 [file pediatrics-v9-e92070-s001.docx]

**Feasibility and Acceptability of Using a Digital Indoor Air Quality Monitor**^a^

|  | | **n (%)** |
| --- | --- | --- |
| **Feasibility Survey Questions (n=15)** | | |
| How often did the child sleep in the same room as the air quality monitor? | | |
|  | Everyday | 15 (100) |
|  | >50% of days in a week (4 or more) | - |
|  | <50% of days in a week (3 or less) | - |
| How would you rate the set-up of the air quality monitor (uHoo- the white device) at home?^b^ | | |
|  | Easy/Very Easy | 15 (100) |
| How would you rate the set-up of the Wi-Fi hotspot at home (the black device?)^b^ | | |
|  | Easy/Very Easy | 15 (100) |
| **Feasibility Interview Findings (n=13)** | | |
| “It was hard to remember like what like if there was like a flashing light I couldn't remember what that meant like if something was wrong or like what it meant.”  -A few of the interviewed participants (n=6) noted that they had to troubleshoot the monitor or were unsure (n=10) what the flashing lights on the monitor indicated and whether they needed to intervene to fix  “Once it was plugged in, it was on autopilot. There's really nothing I had to do."  - All participants reported the set-up of the air monitor at home, and the Wi-Fi mobile hotspot was easy. | | |
| **Acceptability Survey Questions (n=15)** | | |
| How likely are you to use the air quality monitor in the future if this device was provided to you in clinic? ^2^ | | |
|  | Neutral | 2 (13.3) |
|  | Very likely/Likely | 13 (86.6) |
| How likely are you to recommend this technology to a family caring for a child with asthma?^c^ | | |
|  | Neutral | 2 (13.3) |
|  | Very likely/Likely | 13 (86.6) |
| Would you have joined the study if the Wi-Fi hotspot was not provided? | | |
|  | Yes | 11 (73) |
|  | No | 1 (7) |
|  | Not sure | 3 (20) |
| **Acceptability Interview Findings (n=13)** | | |
| “Small enough and it's concise…So you literally could just throw it in a corner...and just let it sit there forever.”  - Most participants described that the monitors did not get in the way of their daily activities (n=10).  “The complaint that my son had was that it blinked a lot so. He has a sensory processing disorder, which means that if he sees something that is just out of sorts, he will look at it and he will touch it.”  - One participant noted that the lights were bothersome to their child with special needs.  “I would love to see where this device goes throughout the summer, and does it change with seasons?... because my child's asthma gets worse when it gets hot, and those season changes like at the beginning of spring.”  - A participant noted they would have liked to see the data collection year-round since their child's symptoms worsened at different times. | | |

^a^ All participants who completed the exit interview (n=13) had corresponding exit survey data.

^b^ Other item responses included *very difficult* and *difficult* or *neutral*

^c^ Other item responses included *very unlikely* and *unlikely*
